# Clinical Outcomes of Critically Ill Patients with *Candida* spp. Peritonitis: A Retrospective Cohort Study

**DOI:** 10.3390/jof11080562

**Published:** 2025-07-29

**Authors:** Gustavo Adolfo González-González, Laura Cristina Nocua-Báez, Sugeich Melendez-Rhenals, Patricia Reyes, Jorge Alberto Cortés

**Affiliations:** 1 Department of Internal Medicine, Faculty of Medicine, Universidad Nacional de Colombia, Bogotá 111321, Colombia; gusagonzalezgon@unal.edu.co (G.A.G.-G.);; 2 Infectious Diseases Department, Hospital Universitario Nacional de Colombia, Office of Clinical Research, Campus Santa Rosa, Bogotá 111321, Colombia; 3 Infectious Diseases Department, Clínica Universitaria Colombia, Clínica Colsanitas, Bogotá 110111, Colombia

**Keywords:** peritonitis, candidiasis, *Candida albicans*, critical care, cohort studies

## Abstract

**Introduction/objectives**: Peritonitis resulting from *Candida* spp. is common among critically ill patients and has been associated with adverse clinical outcomes. This study aimed to determine the effects of isolates of *Candida* species in patients with peritonitis on in-hospital mortality, general hospital stay, and intensive care unit (ICU) stays. **Methods**: This retrospective cohort study was conducted in two highly complex hospitals in Bogotá, Colombia, specifically by reference to patients who were hospitalized in the ICU between 2016 and 2022 with a clinical and microbiological diagnosis of peritonitis. For the analysis conducted for this research, two groups were established: patients with isolates of *Candida* spp. in the peritoneum and patients who had at least one bacterial microorganism in the culture. Multivariate logistic regression models and counting models featuring different mortality outcomes, different lengths of stay in the ICU, and different lengths of stay in the hospital were generated to evaluate the effect of the presence of *Candida* spp. and to account for potentially confounding variables. **Results**: A total of 373 patients, including 83 with *Candida* spp. and 290 with a bacterial etiology, were identified. Among the former group of patients, the most frequently identified species were *C. albicans* (50, 60.2%), *C. tropicalis* (18, 21.7%), and *C. glabrata* (7, 8.4%), whereas among the latter group, *E. coli* (186, 48.5%), *K. pneumoniae* (110, 29.8%), and *E. faecalis* (63, 16.9%) were most frequent. The 30-day mortality rate among patients with peritonitis and *Candida* isolates was 36.1%, and the corresponding rate among patients in the bacterial peritonitis group was 31.4% (*p* = 0.071). After adjustments were made to account for covariates, no significant differences were observed in mortality at 30 days (OR 0.75, 95% CI 0.20–1.18), length of hospital stay (iRR 1.11, 95% CI 0.90–1.40), or length of stay in the ICU (iRR 1.11, 95% CI 0.39) with respect to patients with peritonitis without fungal isolates. The Simplified Acute Physiology Score (SAPS2) (OR 1.04, 95% CI 1.03–1.06), World Society of Emergency Surgery (WSES) score (OR 1.11, (1.03–1.19), previous use of antifungals (OR 2.33, 1.21–4.52), and connective tissue disease (OR 3.71, 95% CI 1.30–10.99) were associated with 30-day mortality. **Conclusions**: The isolation of *Candida* species in peritoneal fluid from critically ill patients with peritonitis was not significantly associated with in-hospital mortality, length of hospital stay, or length of ICU stay after adjustments were made to account for other variables.

## 1. Introduction

Peritoneal candidiasis is a clinical form of invasive candidiasis, and its incidence ranges from 4% to 32% among critically ill patients [1]. The mortality of patients hospitalized in intensive care units (ICUs) with peritonitis as a result of *Candida* species is 41% [1,2]. The main risk factors associated with these infections include mechanical ventilation, a history of bacteremia, immunosuppression, and infectious foci in the upper abdomen [3]. Studies have reported that the majority of such infections occur in the postoperative setting (84%) in relation to anastomotic leaks of multiple origins [4].

The role played by yeast isolation in the peritoneum has not been clearly related to mortality among critically ill patients. Lee et al. reported significant mortality rates in 62 cases of peritonitis among patients with *Candida* peritonitis (21.7%) in comparison with patients with bacterial peritonitis (0%) and those with negative cultures (3.4%), regardless of whether the patients in question received antifungal treatment [5]. In some studies, such as those conducted by Montravers and DuPont et al., among patients with peritonitis associated with health care, the identification of *Candida* has been suggested to constitute an independent risk factor for mortality, with an OR of 3.0 (95% CI 1.3–6.7). However, the size of the samples investigated in those contexts, the imprecision of the relationship between the exposure and the outcome, and the study designs used in such research have limited the extent to which we can be certain of this association [6,7].

This study aimed to identify the effects of isolates of *Candida* species from critically ill patients with peritonitis on the most relevant clinical outcomes, namely, in-hospital mortality rates, hospital stays, and ICU stays, among adult patients who were hospitalized at two tertiary institutions in Bogotá, Colombia.

## 2. Materials and Methods

### 2.1. Stage

This retrospective cohort study focused on the medical records of adult patients who were hospitalized at two third-level institutions (Hospital Universitario Nacional de Colombia, HUN, and Clínica Universitaria Colombia, CUC) in the city of Bogotá. These institutions feature 46 intensive care beds (at HUN) and 49 intensive care beds (at CUC), and they perform highly complex surgical procedures, including abdominal surgery, cardiovascular surgery, cancer treatment, and solid organ transplantation (CUC), among others.

### 2.2. Inclusion and Exclusion Criteria

Adult patients with a clinical and microbiological diagnosis of peritonitis between 2016 and 2022 were included in this research. Only patients with positive microbiological isolation, which was available in the institutional laboratory from peritoneal fluid, were included, regardless of the sampling method used (i.e., surgery or percutaneous puncture with sterile technique). Sampling by the treatment group was performed in accordance with relevant clinical criteria during the evaluation of the patient by surgeons or intensive care physicians. Patients for whom no information regarding the proposed outcomes was available, those whose laboratory isolation reports exhibited inconsistencies, and those who were transferred to another institution were excluded.

### 2.3. Definitions and Outcomes

The definition of peritonitis focused on the evaluation of a microbiological isolate from the peritoneal fluid of a patient with clinical signs of infection and localized abdominal symptoms. The peritonitis type was identified as primary, secondary, or tertiary. The exposed group included patients whose *Candida* species were microbiologically isolated from the peritoneal fluid, whereas the comparison group included patients who exhibited some other type of isolation (bacterial). The primary outcome was in-hospital mortality at 30 days, and the length of hospital stay and length of ICU stay were included as secondary outcomes.

The clinical data of the selected patients were extracted by trained research assistants from an electronic form via the RedCap tool (Vanderbild University, Nashville, TN, USA). The covariates included in the analysis performed for this study were selected from previous studies on mortality among patients with peritoneal candidiasis. These variables included the following: sociodemographic characteristics (age, sex, and hospital); clinical variables, specifically with respect to the most extreme value observed within 24 perioperative hours (temperature, heart rate, inspired oxygen fraction, respiratory rate, systolic blood pressure, and Glasgow score); comorbidities (heart failure, acute myocardial infarction, cerebrovascular disease, peripheral vascular disease, liver disease, connective tissue disease, peptic ulcer, pancreatitis, pneumonia, chronic obstructive pulmonary disease (COPD), chronic kidney disease, use of dialysis, diabetes, dementia, pharmacological immunosuppression, active cancer, hematological neoplasia, metastasis, human immunodeficiency virus (HIV) infection, AIDS, neutropenia, and transplantation); and other variables that have previously been identified as modifiers in this context, as well as the risks associated with the proposed outcomes, such as the organ site of peritonitis (defined in relation to the ligament of Treitz); the presence of intestinal perforation or anastomotic leakage; the number of previous abdominal surgeries; the formulation of antifungals and antimicrobials; and prognostic scores such as the Simplified Acute Physiology Score (SAPS) and the World Society of Emergency Surgery (WSES). Additionally, data concerning the indication for surgical time were obtained with respect to three categories: (1) the surgery was scheduled to occur within the first 24 h of hospitalization; (2) the surgery was scheduled to occur more than 24 h following admission but within the first week of hospitalization; and (3) the surgery was scheduled to occur more than one week following hospitalization.

### 2.4. Procedures

To facilitate the preliminary identification of candidate patients for inclusion in the study, all the final reports of positive peritoneal fluid cultures from 2016 to 2022 were requested from the institutional laboratories. After compliance with the inclusion and exclusion criteria for the study was verified, the data were extracted.

### 2.5. Microbiological Procedures

All the samples were processed in the laboratory associated with the same institution. The Vitek2 system (BioMérieux, Marcy l’Etoile, France) was used in CUC, and subsequent identification was facilitated by mass spectrometry with matrix-assisted laser desorption/ionization and time of flight (MALDI-TOF). At HUN, the BD Phoenix M50 system (Becton, Dickinson and Company, Franklin Lakes, NJ, USA) was used. No routine detection of anaerobic microorganisms was performed.

### 2.6. Statistical Analysis

In the descriptive analysis of the categorical variables, absolute or relative frequencies were used. Continuous variables with normal distributions are presented in terms of means and standard deviations; data with nonnormal distributions are presented in terms of medians and interquartile ranges.

In the in-hospital mortality outcome, a multivariate logistic regression model was used, in which context the dependent variable was in-hospital mortality at 30 days, and the independent variables were selected on the basis of the bivariate analysis with *p* < 0.2. The model that exhibited the lowest amount of error and offered the most information possible was chosen. For the analysis of the length of hospital stay and length of ICU stay, negative binomial models in which the dependent variable was the length of stay and the independent variables were selected considering the bivariate analysis with *p* < 0.2 were used. In the analysis of ICU stay, only the survivors whose stay in the ICU occurred following isolation were included. *p*-values less than 0.05 were considered to indicate statistical significance. An additional exploratory analysis was performed with the patients who died after 30 days (late mortality). For this anaysis a multivariate logistic regression model with the patients who survived the first 30 days was performed.

## 3. Results

**Figure 1:**
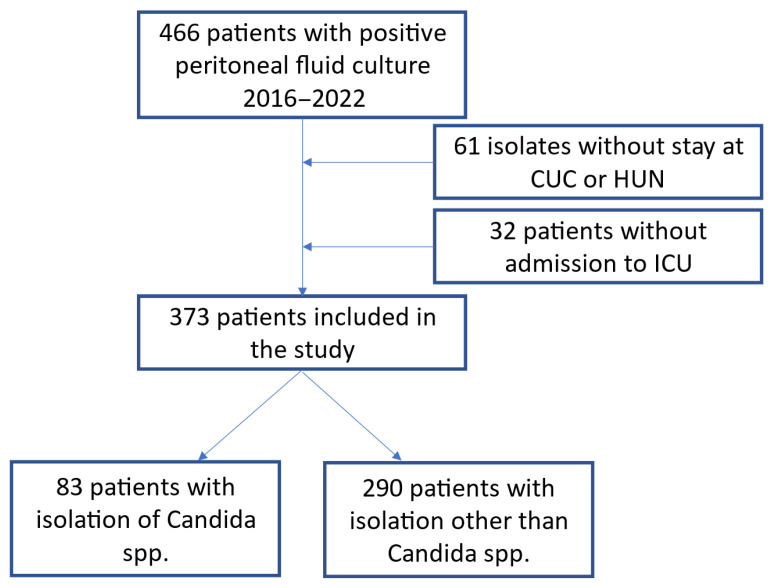
Flow diagram of the patients included in the study.

Between January 2016 and December 2022, 466 patients with positive peritoneal cultures were identified at the two institutions. In total, 93 patients were excluded because of a lack of clinical data (Figure 1), and 373 hospitalized ICU patients with peritonitis were followed as part of this research. The average age of these patients was 60 years (SD: 15.7 years), 68% had secondary peritonitis, 26% had tertiary peritonitis, and 6% had primary peritonitis. The peritoneal fluid samples were extracted during surgery in 85% of the cases, via percutaneous drainage in 12%, and by paracentesis in 3%. A total of 19% percent of the patients had undergone at least one abdominal surgery prior to diagnosis, with an average of 1.7 (SD: 2.1) previous intra-abdominal surgical procedures; such surgeries were more common in the group with the *Candida* isolates (2.4 vs. 1.5, *p* < 0.001); furthermore, intestinal perforation was observed in 189 patients (50.7%).

Among the most common comorbidities, 159 (42.6%) patients had cancer; furthermore, 54 patients (15.7%) had metastasis, 66 (17.7%) had diabetes, and 44 (11.8%) had chronic kidney disease. In total, 41 (11%) patients had peripheral vascular disease, 32 (8.6%) had a history of peptic ulcers, 31 (8.3%) had pancreatitis, and 24 (6.4%) had chronic liver disease. The Charlson score was calculated for all the patients; the mean Charlson score was 4.2 (SD: 3.1) points, and the mean WSES score was 7.2 (SD: 3.7) points. With respect to immunosuppression, a pharmacological cause was identified in 38 (10.2%) patients: 20 had connective tissue diseases, 9 had hematological neoplasms, 3 had HIV infection, 5 had neutropenia, and 1 was a transplant patient. The main demographic characteristics of the participants in this research and the corresponding risk factors are presented in Table 1.

All the patients resided in the ICU during their hospital stay, and 301 patients (80.7%) resided in the ICU at the time of isolation; their average previous stay in the unit before isolation was 5.2 days (SD 9.2). At the time of diagnosis, 46 (12.3%) patients had fever, 77 (20.6%) were hypotensive, and 204 (54.7%) had a history of mechanical ventilation; the average duration of mechanical ventilation prior to isolation was 5.3 days (SD: 8.3). In 354 patients (94.9%), the use of a central venous catheter was identified at the time of diagnosis. The use of parenteral nutrition prior to diagnosis was observed in 136 (36.5%) patients; the use of this method was more frequent in cases involving *Candida* spp. (Table 1). A total of 97.9% of the patients received an antibiotic during their hospitalization in this ICU; however, 46 (12.3%) patients received that antibiotic prior to the diagnosis of peritonitis, and no differences were observed between the study groups in this respect. In total, 103 (27.6%) patients received treatment with antifungal agents prior to *Candida* isolation; this observation was more common in the group with fungal isolates (51.8% vs. 20.7%, *p* < 0.001).

### 3.1. Microbiological Findings

The microbiological findings concerning the included patients with *Candida* peritonitis (n = 83) are presented in Table 2; in 62 (74.7%) of these patients, at least one aerobic bacterial isolate was observed. In total, only 21 patients (accounting for 25.3% of those with a *Candida* isolate) had a single infectious agent isolated. In this group, it was more common for a single accompanying bacterial microorganism to be observed than was the case in the group with bacterial infection (37.3% vs. 31% <0.001). Microbiological data obtained from the patients with bacterial infections are presented in Table 2. On average, a greater number of bacterial microorganisms were observed for each culture in the peritoneum in the group with bacterial infection than in the group with fungal isolates (1.4, SD 0.9, vs. 1.1, SD 1.0, *p* = 0.05).

### 3.2. Treatment and Outcomes

In total, 175 patients, including 109 (37.6%) in the bacterial peritonitis group and 66 (79.5%) in the fungal isolation group, received an antifungal agent. The use of both empirical and targeted antifungals was more common in the group with intra-abdominal *Candida* isolates (79.5% vs. 37.6%, *p* < 0.001), and the duration of therapy was longer in this case (15.7 days vs. 5.8 days, *p* < 0.001). A total of 65 patients (78.3%) in the fungal infection group received empirical antifungal agents, whereas 106 patients (36.6%) in the bacterial infection group received antifungal agents. Among the empirical antifungals used in this context, fluconazole, which was used in 40 patients (48.2%) in the fungal infection group and in 66 patients (22.8%) in the bacterial infection group, was the most common, followed by caspofungin, which was used in 24 patients (28.9%) in the group with fungal infection and in 40 patients (13.8%) in the group with bacterial isolates (*p* < 0.001); only 1 patient was managed with liposomal amphotericin. Antifungal changes were observed in 21 (25.3%) of the patients with fungal peritonitis and in 18 (6.2%) of the patients with bacterial peritonitis (*p* < 0.001), in which context caspofungin was the most frequent second-line treatment (accounting for 15 patients in the fungal infection group, 18.1%, and 15 patients in the bacterial infection group, 5.2%; *p* < 0.001), followed by fluconazole (17 patients). In this context, seven patients were treated with amphotericin B, and one patient was treated with anidulafungin. The most frequent changes were observed in patients with isolates of *C. albicans* (six cases toward caspofungin) and *C. tropicalis* (six cases toward caspofungin), although this situation was not observed in the case of *N. glabratus* and, in that of *C. auris*, a switch to liposomal amphotericin was made.

**Figure 2:**
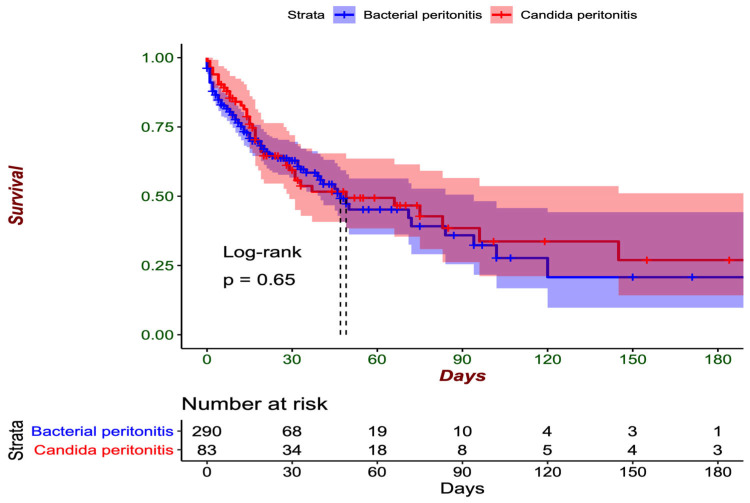
Thirty-day survival curves (Kaplan-Meier) of the peritoneal infection groups.

In both groups, the presence of multiorgan dysfunction, the need for vasopressors, the creatinine values, and the development of acute kidney injury were similar. The group with fungal peritonitis had a systemic inflammatory syndrome in 61 cases (73.5%) vs. 209 cases in the other group (71.8%, *p* = 0.87), creatinine levels had a mean value of 1.41 mg/dL vs. 1.8 mg/dL (*p* = 0.10) On average, the patients were hospitalized for 11.4 days (SD: 14.6) before the diagnosis of peritonitis. Hospital mortality was greater among the patients with fungal isolates in the peritoneum than among those with only bacterial isolates (49.4% vs. 37.6%, *p* = 0.071) as shown in Figure 2, and the time to death was greater in the group with intraperitoneal *Candida* (40.6 days, SD: 50.6 vs. 22.4 days, SD: 26.0 days, *p* < 0.001).

When mortality was evaluated at 30 days of hospitalization, similar outcomes were observed between the patients with mycotic isolation and those with only bacterial isolation (36.1% vs. 31.4%, *p* = 0.494), as shown in Figure 2. Within the group of patients with fungal isolates in the peritoneum, the highest number of cases of mortality at 30 days was observed among patients in whom *C. albicans* was isolated in 19 cases (38%), followed by *C. tropicalis* in six cases (33%), *C. glabrata* in two cases (29%), and *C. parapsilosis* in two cases (50%). The only patient in whom *C. lusitaniae* was isolated died within the first 30 days of the hospital stay. In the group with exclusively bacterial isolates, the highest number of deaths within the first 30 days was observed among individuals with *E. coli* isolation (59 cases, 33%), followed by *K. pneumoniae* (40 cases, 36%) and *E. faecalis* (20 cases, 31%).

### 3.3. Risk Factors for Mortality

Following the analysis of the multivariate logistic regression model, no significant differences were observed between the presence of *Candida* species in the peritoneal fluid or sample and mortality (OR 0.75, 95% CI 0.20–1.18). Other variables were significantly associated with increased 30-day mortality (Table 3). Previous antifungal use was associated with mortality (accounting for 24.0% of patients who died vs. 12.3% of those who survived, *p* = 0.045), whereas empirical use was not associated with mortality (32.2% mortality in patients who received empirical antifungal agents vs. 30.2% of those who survived, *p* = 0.775). A sensitivity analysis was conducted to evaluate the possible modifying effects or interactions between previous antifungal use and empirical antifungal use. No associations were observed between these variables and the primary outcome, and their presence did not significantly modify the estimator of the relationship between peritonitis with fungal isolation and 30-day mortality (OR 0.81; 95% CI 0.38–1.73). Risk factors for late mortality included multiorganic dysfuntion (OR 48; 95% CI: 7–1018), antifungal use (OR 18.95; 95% CI 2.94–383), *K. pneumoniae* identification (OR 5.17; 95% CI 1.62–18.62), mechanical ventilation duration (per day, OR 1.12; 95% CI 1.06–1.22). The isolation of *Candida* spp. was not associated with an effect on mortality (OR 1.34; 95% CI 0.39–4.63).

### 3.4. Risk Factors for Hospital Stays

Among the secondary outcomes, isolation of *Candida* from the peritoneum was not significantly associated with hospital stay following diagnosis (iRR 1.11, 95% CI 0.90–1.40). However, multiorgan dysfunction (iRR 1.2, 95% CI 1.00–1.45), parenteral nutrition (iRR 1.44, 95% CI 1.20–1.74), time spent undergoing mechanical ventilation (iRR 1.03, 95% CI 1.02–1.05), having required a change in antifungals (iRR 1.57, 95% CI 1.30–1.90), having experienced an anastomotic leak (iRR 1.48, 95% CI 1.23–1.79), and the use of a central catheter (iRR 1.61, 95% CI 1.13–2.31) were significantly related to this factor.

### 3.5. Risk Factors for ICU Stays

In the analysis of this outcome, only surviving patients whose microbiological isolation occurred with an active stay in the ICU were included; in total, 172 such patients were identified, including 26 from the group with fungal isolates and 146 in the group without fungal isolates. Prior to the adjustment of the model, large differences in the average length of stay in the ICU following isolation were observed between the two groups (15.19 vs. 7.86 days). When the negative binomial model was used, exposure was not significantly related to this outcome (iRR 1.11, 95% CI 0.88–1.39). On the other hand, factors such as a history of previous abdominal surgery (iRR 1.29, 95% CI 1.04–1.62), duration of mechanical ventilation (iRR 1.06, 95% CI 1.05–1.07), length of stay in the ICU prior to isolation (iRR 1.09, 95% CI 1.08–1.11), use of empirical antifungals (iRR 1.39, 95% CI 1.15–1.70), and history of acute kidney injury (iRR 1.35, 95% CI 1.13–1.61) were significantly related to this outcome.

## 4. Discussion

Our study revealed that in a retrospective cohort of critically ill patients with peritonitis in two highly complex hospitals in Bogotá, Colombia, the isolation of *Candida* species in peritoneal fluid via surgical procedures or percutaneous drainage was not significantly associated with mortality; furthermore, this factor was not significantly associated with general hospital stays or stays in the ICU. The use of antifungals also did not have a favorable effect on this group of patients.

This finding is consistent with the results reported by Montravers et al. in 2017 by reference to a retrospective cohort of patients with suspected *Candida* peritonitis [8], in whom the presence of fungal isolates was not related to mortality in comparison with patients in whom fungal infection was not confirmed. Similar mortality rates were observed in patients in whom a *Candida* species was identified in the peritoneum in comparison with patients who did not have a fungal infection or who had such an infection in another location (OR 0.84, 95% CI 0.48–1.50). Similarly, in a retrospective study of patients with culture-confirmed peritonitis conducted in Turkey, the isolation of fungal species was not significantly correlated with increased 30-day mortality (10.6% vs. 4.1%, *p* = 0.202) [9]. However, contradictory results have been reported concerning the impact of the isolation of *Candida* from the peritoneum. In a previous study, Montravers et al. identified the isolation of these microorganisms as an independent risk factor for mortality (OR 3.0, 95% CI 1.3–6.7; *p* < 0.01) among patients with hospital-acquired peritonitis. These authors reported higher mortality among patients with such isolation than among those without such isolation. The difference in these results can be explained by the different types of studies conducted in this context. This study conducted by Montravers et al. featured an asynchronous case—control design over time and excluded patients with pancreatitis and hollow viscus perforation [7]. Case—control studies are limited in terms of their ability to control for selection biases introduced by the population chosen for the study. The HELP (HELP = HEaLthcare-associated intra-abdominal infection study in critically ill Patients) study, a prospective, observational, and multicenter study that was conducted in 17 ICUs associated with university hospitals in Spain and that focused on patients with peritonitis, revealed that the isolation of *Candida* was significantly associated with 30-day mortality (OR 3.05, 95% CI 1.18–7.87; *p* = 0.022) [10]. However, this study included patients who exhibited intra-abdominal pathologies other than peritonitis, thus introducing the possibility of negative cultures; furthermore, in its multivariate analysis, it excluded patients who were considered to be immunosuppressed (i.e., users of chemotherapy or prolonged steroids, transplants, or living with HIV or immunosuppressants). In contrast to the results of our study, 30-day mortality was reported to be lower in patients with intra-abdominal infection (14.5%). Owing to the study design, the claim that the clinical severity, as measured by prognostic scores, was greater in deceased patients with mycotic isolates cannot be confirmed. Given the ongoing controversy in this context and the lack of clear evidence indicating the benefits of administering antifungal drugs to this group of patients, we maintain that the conditions necessary to conduct a controlled clinical trial to clarify this problem are met in this context.

Regarding the use of antifungals among patients with peritonitis, in our study, administration prior to diagnosis was significantly related to increased mortality, whereas the empirical or direct use of this approach did not modify this outcome. According to a retrospective cohort study matched by propensity score in Taiwan, which was performed by reference to patients with peritonitis secondary to perforated peptic ulcers with fungal isolates, no significant differences were observed in mortality (15% vs. 10% *p* = 0.45), prolonged ICU stay (10% vs. 27.5% *p* = 0.05), or prolonged mechanical ventilation (7.5% vs. 20% *p* = 0.11) between patients who received antifungal therapy and those who did not [11]. There is also information that pharmacokinetic and pharmacodynamic considerations should be held in mind when approaching patients with fungal infection of the peritoneum [12]. Furthermore, a multicenter study in Europe identified the use of antifungals as a risk factor for the development of intra-abdominal candidiasis [13]. We maintain that it is possible that antifungal use prior to diagnosis is motivated more strongly by the severity of the patient’s condition than by microbiological findings. In another study conducted by Lichtenstern et al. among patients with fungal isolates resulting from sepsis or septic shock, the use of antifungal drugs did not affect mortality (OR 0.92, 95% CI 0.68–1.26) [14]. Similarly, a retrospective cohort study conducted in Brazil to investigate oncology patients with microbiological confirmation of intra-abdominal candidiasis revealed that the prescription of antifungals did not significantly impact 30-day mortality (71.1% vs. 69.8%, *p* = 0.788) [15]. Data obtained from a small retrospective study of patients with various forms of intra-abdominal candidiasis in China, which exhibited possible selection biases according to the type of patients included, revealed that an appropriate initial antifungal therapy strategy (initiated within the first 5 days of culture for a subsequently susceptible microorganism, specifically at the optimal dose) had a favorable impact on mortality (HR 0.18, 95% CI, 0.07–0.47) [16].

The 2024 global guidelines for patients with intra-abdominal infections highlight the importance of considering the use of early antifungal agents among critically ill or immunosuppressed patients with community-acquired infections [17]. Similarly, the guidelines provided by the Infectious Diseases Society of the United States (IDSA) suggest that empirical antifungal therapy should be considered for patients with clinical evidence of intra-abdominal infection and significant risk factors for candidiasis [18]. The evidence collected in this and other studies suggests that the benefit of using antifungals among patients with isolates of *Candida* species in the peritoneum is unclear; this approach is the only strategy that aims to mitigate the morbidity and mortality of this fungal infection, and so a randomized clinical trial is needed in cases involving high-risk patients (as evaluated by WSES and SAPS2) with multiorgan involvement; this process involves evaluating the early and unified initiation of broad-spectrum antifungals among these groups of patients while adjusting for focus control. To date, no benefits resulting from the use of a single antifungal have been identified, and the evidence indicates that this approach is used only as a result of the fear of poor outcomes among high-risk patients.

In this cohort of patients, the WSES and SAPS2 severity scores of peritonitis were related to mortality. In a study conducted by Li WS et al. in Taiwan, clinical severity, as measured via the Acute Physiology and Chronic Health Evaluation (APACHE) score (>20 points), was identified as an independent risk factor for mortality (OR 9.5, 95% CI 1.1–80.7; *p* = 0.04) [11]. De Almeida BL et al. reported similar results by reference to a specific group of patients with peritoneal candidiasis and abdominal neoplasms; however, the Sequential Organ Failure Assessment (SOFA) score was used in this context (OR = 1.30, 95% CI 1.094–1.562; *p* = 0.003) [15]. Maseda et al. reported that SAPS2 was also associated with an increased risk of mortality in patients with peritonitis, including those with a fungal etiology (OR 1.08, 95% CI 1.05–1.11; *p* <0.001) [10]. In the AmarCAND2 cohort, Leroy et al. reported that a SAPS> 46 was significantly associated with mortality, with an OR of 2.89 (95% CI 1.81–4.63; *p* <0.001). Notably, in this cohort, immunosuppression, in which context not only autoimmune diseases but also immunosuppressive drugs and congenital and acquired diseases were considered, also exhibited a significant OR of 1.98 (95% CI 1.03–3.80) with respect to mortality [19]. This information indicates that the severity of the organic compromise in this infection is an independent variable that is associated with mortality, for which the strategies used to identify early diagnosis and resuscitation for patients should be promoted and emphasized because this approach is likely to mitigate morbidity and mortality, especially among immunocompromised patients.

We reported a general mortality rate that was similar to the rates that have been described in other contexts, at approximately 40% [20]. The microbiological findings of this research also suggest that the most frequently isolated species is *C. albicans;* however, in our study, the species *C. tropicalis*, *C. glabrata*, and *C. parapsilosis* were more prevalent. Unlike the results that have been reported in the French cohort, the microorganisms that followed *C. albicans* in terms of frequency were *C. glabrata*, *C. krusei*, and *C. kefyr* [21]. In this study, although mortality notably did not change according to the isolated species, the establishment of an association in this context was not feasible because of the limited number of cases of each of the species.

In this study, the isolation of *Candida* from the peritoneum in patients with peritonitis was not significantly associated with the patients’ length of hospital stay; however, consistent with the findings presented above, factors pertaining to a deterioration in the clinical state, such as presenting multiorgan dysfunction, longer durations of mechanical ventilation and parenteral nutrition, were significantly related to this outcome. Notably, variables such as the use of parenteral nutrition and clinical severity were identified as risk factors for invasive candidiasis within the consensus of the ÉPICO project for the management of patients with suspected intra-abdominal infection [22].

Among the limitations of the study, the risk of selection bias that is inherent in the retrospective nature of this research is notable. It is possible that in patients with multiple peritoneal interventions, the search for fungal species may not have been conducted in all cases, which could cause the number of cases of peritonitis with this isolation to be underestimated. To reduce the occurrence of this bias, only patients with positive cultures were included in this research. It was not possible to determine the antifungal sensitivity of the isolates, which did not allow the researchers to evaluate the adequate formulation and its respective impact; furthermore, it was not possible to identify the extent to which the infectious focus was controlled following the diagnosis of peritonitis, which could impact patient outcomes.

An important limitation could be derived from the lack of identification of anaerobic microorganisms, which can also play a significant role in peritonitis. Among the most recognized is *Bacteroides fragilis*, though a wide variety of other organisms, such as *Clostridiodes* spp., *Streptococci*, and even *Lactobacillus* spp., have been implicated in the etiology of this infection in up to two-thirds of cases, particularly in the context of secondary peritonitis due to distal bowel perforation or obstruction [23]. Although some studies on the microbiology of peritonitis report a low frequency of anaerobes, this may be attributed to the challenges of microbiological identification when compared to aerobes, as anaerobic organisms require specific sampling conditions, specialized culture media, prolonged incubation times, and strict oxygen-free environments [24]. Our study did not perform routine identification of anaerobic organisms, which may have contributed to the underreporting of mixed infections. The impact of the anaerobes on mortality in patients with peritonitis due to bacterial or fungal infections is not clear, since most of the time the empirical treatment includes antibiotics that cover anaerobes. A previous study has found that anaerobes might be more frequently found among patients with shock in this group of patients [25].

## 5. Conclusions

This study revealed that isolation of *Candida* species in peritoneal fluid from patients with peritonitis who were hospitalized in ICUs was not significantly associated with in-hospital mortality, hospital stay, or length of stay in the ICU. As expected, the SAPS2 and WSES clinical severity scores have an important impact on mortality, just as previous antifungal use and a history of connective tissue disease, which were significantly associated with 30-day mortality in this context.

## Figures and Tables

**Table 1:** Basic clinical and sociodemographic characteristics of the patients.

Variables	Isolation of *Candida* spp. in Peritoneal Fluid		p
	No (n = 290)	Yes (n = 83)	
Age, mean (SD)	59.75 (15.6)	60.72 (15.9)	0.618
Male sex, n (%)	156 (53.8)	36 (43.4)	0.121
SIRS, yes, n (%)	208 (71.7)	61 (73.5)	0.859
Type of peritonitis			<0.001
Primary, n (%)	19 (6.6)	5 (6.0)	
Secondary, n (%)	212 (73.1)	42 (50.6)	
Tertiary, n (%)	59 (20.3)	36 (43.4)	
Location of origin of peritonitis			0.073
Indefinite, n (%)	26 (9.0)	5 (6.0)	
Lower gastrointestinal tract, n (%)	229 (79.0)	60 (72.3)	
Upper gastrointestinal tract, n (%)	35 (12.1)	18 (21.7)	
Type of surgical programming			0.844
Surgery within the first 24 h of stay, n (%)	191 (65.9)	57 (68.7)	
Surgery between days 2–7 days of stay, n (%)	57 (19.7)	14 (16.9)	
Surgery following 1st week of stay, n (%)	42 (14.5)	12 (14.5)	
Abdominal surgery prior to isolation, yes, n (%)	221 (76.2)	74 (89.2)	0.016
Number of previous abdominal surgeries, mean (SD)	1.53 (1.8)	2.42 (2.6)	<0.001
Dialysis, yes, n (%)	23 (7.9)	22 (26.5)	<0.001
Previous antibiotic use, yes, n (%)	37 (12.8)	9 (10.8)	0.781
SOFA score, mean (SD)	5.16 (3.6)	6.10 (4.2)	0.044
SAPS2 score, mean (SD)	34.17 (16.6)	39.33 (17.4)	0.014
WSES score, mean (SD)	7.14 (3.8)	7.59 (3.3)	0.329
Charlson score, mean (SD)	4.19 (3.1)	4.45 (3.1)	0.506
Previous mechanical ventilation, yes, n (%)	149 (51.4)	55 (66.3)	0.023
Duration of previous mechanical ventilation, mean (SD)	4.74 (7.8)	7.43 (9.6)	0.009
Previous parenteral nutrition, yes, n (%)	87 (30.0)	49 (59.0)	<0.001
ICU stay prior to peritonitis, yes, n (%)	264 (91.0)	78 (94.0)	0.528
Previous ICU time, mean (SD)	4.93 (9.8)	5.99 (6.5)	0.354

SIRS: systemic inflammatory response syndrome; SD: standard deviation. SAPS2: Simplified Acute Physiology Score 2; SOFA: Sequential Organ Failure Assessment; WSES: World Society of Emergency Surgery;

**Table 2:** Description of microbiological isolates in patients with peritonitis and identification of fungus.

Peritonitis with Isolates of *Candida* spp. n = 83 (%)			Bacterial Peritonitis n = 290 (%)	
Only *Candida* spp. n = 21 (25.3)	*Candida albicans*	15	*Escherichia coli*	155 (53.1)
	*Candida tropicalis*	5	*Klebsiella pneumoniae*	93 (32.1)
	*Nakaseomyces grabratus*	1	*Enterococcus faecalis*	47 (16.2)
*Candida albicans* n = 50 (60.2)	Only *C. albicans*	15	*Pseudomonas aeruginosa*	37 (12.8)
	*Escherichia coli*	15	*Enterococcus faecium*	33 (11.4)
	*Klebsiella pneumoniae*	9	*Proteus mirabilis*	20 (6.9)
	*Pseudomonas aeruginosa*	8	*Staphylococcus aureus*	7 (2.4)
	*Enterococcus faecalis*	6	*Streptococcus anginosus*	4 (1.4)
	*Enterococcus faecium*	6	Other *Streptococcus*	5 (1.7)
*Candida tropicalis* n = 18 (21.7)	Only *C. tropicalis*	5		
	*Escherichia coli*	5		
	*Klebsiella pneumoniae*	5		
	*Pseudomonas aeruginosa*	4		
	*Enterococcus faecalis*	4		
	*Enterococcus faecium*	2		
*Nakaseomyces glabratus* *(C. glabrata)* n = 7 (8.4)	*Escherichia coli*	4		
	*Enterococcus faecalis*	2		
	*Klebsiella pneumoniae*	1		
	*Pseudomonas aeruginosa*	1		
	Only *N. glabratus*	1		
*Candida parapsilosis* n = 4 (4.8)	*Klebsiella pneumoniae*	2		
	*Escherichia coli*	1		
	*Enterococcus faecalis*	1		
*Candidozyma auris* n = 1 (1.2)	*Enterococcus faecalis, Proteus mirabilis*	1		
*Kluyveromyces marxianus* (*C. kefyr)* n = 1 (1.2)	*Enterococcus faecalis, Escherichia coli*	1		
*Clavirospora lusitaniae* (*Candida lusitaniae*) n = 1 (1.2)	*Pseudomonas aeruginosa*	1		
*Pichia kudriavzevii* (*C. krusei)* n = 1 (1.2)	*Enterococcus faecalis, Escherichia coli, Pseudomonas aeruginosa*	1		

**Table 3:** Variables associated with 30-day mortality in the cohort of patients with peritonitis.

Variable	OR *	Lower Limit IC **	Upper Limit IC **
Exhibition	0.88	0.48	1.58
History of mechanical ventilation	1.73	1.00	3.03
Connective tissue disease	3.71	1.30	10.99
SAPS2 score	1.04	1.03	1.06
WSES score	1.11	1.03	1.19
Previous use of antifungals	2.33	1.21	4.52

**: confidence interval; *: odds ratio. SAPS2: Simplified Acute Physiology Score 2; WSES: World Society of Emergency Surgery.

## Data Availability

Anonymized data are available upon request.

## Abbreviations

The following abbreviations are used in this manuscript:

ICU	Intensive Care Unit
SAPS2	Simplified Acute Physiology Score 2
WSES	World Society of Emergency Surgery Sepsis Severity Score
AF	Antifungal
